# Nursing students’ transfer of learning outcomes from simulation-based training to clinical practice: a focus-group study

**DOI:** 10.1186/s12912-019-0376-5

**Published:** 2019-11-08

**Authors:** Jørn Hustad, Berit Johannesen, Mariann Fossum, Olav Johannes Hovland

**Affiliations:** 1 Department of Health and Nursing Science, Faculty of Health and Sport Sciences, University of Agder, Kristiansand, Norway; 2 Department of Health and Nursing Science, Faculty of Health and Sport Sciences, University of Agder, Grimstad, Norway

**Keywords:** High-fidelity simulation training, Nursing students, Nursing education, Thematic analysis

## Abstract

**Background:**

Simulation-based training is used to develop nursing students’ clinical performance in assessing and managing situations in clinical placements. The use of simulation-based training has increased and become an integrated part of nursing education. The aim of this study was to explore nursing students’ experiences of simulation-based training and how the students perceived the transfer of learning to clinical practice.

**Methods:**

Eight focus group interviews were conducted with a total of 32 s- and third-year nursing students who participated in a simulation-based training organized as preparation for clinical placement. The transcribed interviews were analysed with thematic analysis.

**Results:**

Three major themes emerged from the focus group interviews; first, the simulation-based training promoted self-confidence; second, understanding from simulation-based training improved clinical skills and judgements in clinical practice; and third, simulation-based training emphasised the importance of communication and team collaboration.

**Conclusions:**

This study revealed students’ transfer of learning outcomes from simulation-based training to clinical practice. The students’ experiences of the simulation-based training remain as enduring and conscious learning outcomes throughout their completion of clinical practice. The organisation of simulation-based training and its implementation in the curriculum are crucial for the learning outcomes and for students’ experiences of the transfer of knowledge to clinical practice.

## Background

Simulation-based training can be used to model clinical events in a safe environment, thus developing nursing students’ clinical skills in critical thinking, problem solving, decision-making and interdisciplinary collaboration in clinical placements [1]. The use of simulation-based training has increased to become an integrated part of nursing education, and it is recognized as being a beneficial training method [2–4]. Simulation-based training is an effective strategy to increase students’ knowledge, clinical judgement and communication skills [5–7]. Studies have shown promising results from implementing more simulation-based training in nursing education [3, 8, 9]. It is becoming increasingly costly to provide staff and equipment for high-fidelity simulation sessions, which makes it important to investigate how simulation-based training increases students’ self-confidence and learning outcomes. The potential of simulation to help students improve their assessment and management skills in clinical practice should also be studied [8]. However, the literature emphasizes that the learning environment and the equipment need to be realistic and authentic [10]. A lack of authenticity in simulation-based training can decrease the transference of skills to clinical practice [11]. Pre-briefing and debriefing are also crucial to achieving the best learning outcomes in simulation [12, 13]. In a mixed method study with 214 nursing students, increased levels of confidence, together with decision making skills, interprofessional communication, and level of preparedness were the essential competencies necessary for the transition to practice [14]. The design of the simulation program, as well as how academic staff and staff in clinical practice provide metacognitive guidance in debriefing and clinical settings, have an impact on how effectively learning is transferred from simulation-based training to clinical practice [15]. The aim of this study was to explore nursing students’ experiences of simulation-based training and how they perceived the transfer of learning to clinical practice.

## Methods

### Design

A qualitative descriptive design with focus group interviews was adopted. A focus group interview facilitates a group process that can help the participants to clarify and explain their experiences, which would be less accessible in individual interviews. The focus group interview method is suitable when participants have a common background [16, 17].

### Sample and setting

Purposive sampling was employed. A total of 285 nursing students in their second or third year of the bachelor program in nursing at a Norweigan University received an invitation from their course leader to participate in focus group interviews. The students who wanted to participate signed a written consent. The participants were invited to the interviews by email from the researchers. The interviews took place in a room at the university. Thirty-two students (27 females) signed a written consent and were interviewed in eight focus groups of between three and six participants. The characteristics of the participants are displayed in Table 1. The high-fidelity simulation sessions using Laerdal SimMan 3G manikins were mandatory preparation for the students’ hospital clinical practice. The simulation sessions were part of a one-week program and conducted before the students entered their clinical practice. The program included ten e-learning courses provided by the local hospitals, training with technical medical equipment, performing and recording case-scenarios and two acute simulation scenarios. Before the program, the students received extensive written and oral information in class and on the learning platform. The briefing included clarifying course objectives, environment, responsibilities, roles, expectations, logistics, and presentation of the manikins, equipment and communication tools. The de-briefing was conducted according to Gibbs’s reflective cycle, undergoing description, feelings, evaluation, analysis, conclusion and action plan [18]. The operators and facilitators were experienced and formally educated in facilitating. Two high-fidelity scenarios focusing on deteriorating patients were employed. One scenario was about heart disease, and the other was about post-operative bleeding. Some groups performed the scenarios once, other groups performed them twice because the facilitators had different arrangements. Most of the participants took an active role in the scenarios, but a few chose only to observe.

**Table 1:** Demographic characteristics of the participants (*N* = 32)

Characteristics		Number (percent)
Gender	Female	27 (84%)
	Male	4 (16%)
Age	21 years	5 (16%)
	22 years	8 (25%)
	23 years	6 (19%)
	24 years	4 (13%)
	25 years	2 (6%)
	26–33 years	4 (13%)
	Above 34 years	3 (9%)
Study year in the Bachelor program in nursing	Second year	22 (69%)
	Third year	10 (31%)
Types of clinical placement finished before the simulation session (one participant has finished several different clinical placements).	Nursing Home	32 (100%)
	Community Care	10 (31%)
	Mental Health	9 (28%)
	Medical Ward	22 (69%)
	Surgical Ward	21 (66%)

### Data collection

The focus group interviews were conducted in May 2016, October 2016 and February 2017. To approach the students’ perceived transfer of learning outcomes from the simulation-based training to clinical practice, an interview guide was developed with three open ended main questions: What were your experiences of simulation-based training? Second, what were your learning outcomes from the debriefing, and the last question was how has the simulation impacted your practice in the clinical placement? The focus groups were conducted by one of the authors between two and 4 months after the simulation sessions, and the students had performed at least 8 weeks of their clinical studies before they participated in the focus group interview. The focus groups were moderated by the first author (JH), and the last author facilitated and took notes (OJH). The focus group interviews lasted between 42 and 87 min and were audio recorded and transcribed verbatim.

### Data analysis

The demographic data were analysed using descriptive statistics, and numbers and percentages were calculated. The transcribed interview material was thematically analysed by the team of authors, inspired by Braun and Clarke’s six step-by-step guide for thematic analysis [19]. The first step involved reading the transcriptions and becoming familiar with the data. All authors read through the transcriptions to obtain an overview. The next step was to generate initial codes. The first author systematized the material, searching for potential themes using NVivo 11®. Then, all the authors searched for themes and repeatedly reviewed the themes in relation to the coded extracts and the entire data set. The authors met several times and discussed the coding process to finalize the initial sub-themes. The authors then revised, defined and named the themes; examples of the coding are displayed in Table 2.

**Table 2:** Examples of sub-themes and themes from the thematic analysis of the focus group interviews about the nursing students’ experiences with simulation-based training

Example of text coded	Sub-theme	Theme
I had a very negative self-image in the situation. Afterwards they told me that I saved the patient…. My self-confidence increased after having talked together. I could see that I did something correct after all (No. 6).	Positive feedback reduces self-criticism	Simulation-based training promotes self-confidence
I check the symptoms we learned during simulation every day. The skin, the lips – are they blue? Checking breathing and pulse…Knowing what these symptoms mean and what is important to do as the next step. It was useful to learn during simulation (No. 8).	Applying knowledge to the performance of activities	Understanding from simulation-based training improves clinical skills and judgement in practice
In clinical practice, you see that many people communicate unclearly, and that makes you more observant to use closed-loop communication, like we did during simulation (No. 1).	Using communication tools improves communication	Simulation-based training emphasises the importance of communication and team collaboration

## Results

Three themes emerged from the data analysis. (1) Simulation-based training promotes self-confidence, (2) Understanding from simulation-based training improves clinical skills and judgement in clinical practice, and (3) Simulation-based training emphasises the importance of communication and team collaboration. Table 3 displays the themes and sub-themes. We elaborate further on these findings below. The quotes are numbered according to focus group interviews 1–8.

**Table 3:** Overview over the three main themes and sub-themes of nursing students’ experiences with the transfer of learning outcomes from simulation-based training to clinical placements

Themes and sub-themes		
Simulation-based training promoted self-confidence	Understanding from simulation-based training improves clinical skills and judgements in practice	Simulation-based training emphasises the importance of communication and team cooperation
Positive feedback reduces self-criticism Becoming aware of own reactions and behaviour Positive change of attitude	Applying knowledge to the performance of activities Assessing clinical changes Using decision-making tools Discovering practical challenges Developing technical skills	Using communication tools improves communication Experiencing nurse responsibility and the importance of leadership skills Becoming aware of communication with the patient’s relatives

### Simulation-based training promotes self-confidence

Several participants expressed that high-fidelity simulation-based training prepared them mentally for their clinical studies. Some participants described how they experienced stress during the simulation; they expressed self-criticism and explained how they were not initially satisfied with their own achievements. During debriefing they perceived positive responses from the facilitator and peers. One participant said: “The way the teachers communicated with us… They pointed out some mistakes and suggested improvements… They did it in a friendly way, so you didn’t feel stupid… The teachers were very pedagogic, and that resulted in a great learning outcome. It was fantastic for me” (No. 8).

Furthermore, the participants described how they became aware of their own reactions to stress and how other students assessed their behaviour. One participant described how she perceived the feedback: “It is very good to get feedback. You feel that you made many mistakes because you were stressed. But the peers responded and said; you looked so calm and you did everything correct. Knowing that even if it’s chaotic in my head and I am so stressed, I appear calm to others” (No. 3). After they had performed in the simulation-based training scenarios, several of the participants reported that their self-confidence increased before they entered their clinical placements. In particular, the participants who had active nurse roles during the simulation scenarios reported improved learning outcomes. One participant said: “It strengthens your self-confidence before the clinical placement, you don’t feel totally unexperienced… You feel that you have done many things correct. You learn how to do things correct, and you learned from your mistakes in the simulation sessions” (No. 6).

Several participants had received responses from peers that made them reconsider their experiences in a positive manner. One participant said: “Receiving feedback and counselling from peers was an experience of ‘tailwind’ before entering clinical placement” (No. 8).

### Understanding from simulation-based training improves clinical skills and judgement in practice

Participants reported that they prepared thoroughly before the simulation-based training, but they found it difficult to apply this knowledge to the activities they performed during the simulation-based training. They experienced a gap between their theoretical knowledge and actually transforming that knowledge into clinical skills. One said: “The step from theory in books and tasks to hospital reality is overwhelming. Simulation made that step easier to take” (No. 8). They described their experiences from the simulation-based training as very helpful for transferring their knowledge and performing in similar patient situations during clinical practice.

Through simulation-based training, participants became more aware of the importance of examining clinical symptoms and vital signs. One participant said: “Observing all these vital signs, that is what I have brought with me from that case; continually observe and check out: Is it bleeding? Is it infection? How is the skin? Is it cold, clammy, warm”? (No. 6). Participants reported that they had incorporated decision-making tools for evaluating vital signs after having employed those tools in the scenarios for identifying conditions and assessing treatment options. One participant stated: “A patient got acute respiratory problems, so I assessed the patient using the decision-making tools we learned to use during the simulation sessions, and then I contacted the nurse and the doctor. I’ve learned not just to stand there in panic. I felt confident, I know what to do. Without the simulation-based training, I would not dare to act in this situation” (No. 8).

The participants explained that during simulation, they developed important clinical skills that they needed before clinical practice. As one participant expressed, “I was much more prepared, more self-confident, having learned the various kinds of modern equipment, how they work and how I should handle them” (No. 3). Participants also detected many unexpected practical challenges during the scenarios. As one participant described, “We have to give the drug intravenously because the blood pressure is decreasing. Then, you discover that the patient does not have vein cannula. Can we give it orally? Will it have an effect at all? Will it be too late? These things that you do not think of in a lecture lesson – they really hit you during simulation. Then, you realize what you don’t know… It is very useful” (No. 1). Students reported that it was valuable to identify these challenges before entering clinical practice.

### Simulation emphasises the importance of communication and team cooperation

Some participants expressed that during the simulation, they became more aware of the importance of communication and interaction among healthcare professionals. One participant said: “I felt that the communication part is what I learned most from… That is a major part of the care given to a patient; to communicate with your colleagues and present the tasks properly” (No. 5).

In class and through communication exercises, the participants had learned about the importance of communication. Using communication tools in a simulation context increased their understanding of the importance of efficient communication to ensure optimal treatment of the patient, especially in emergency situations. Through the simulation, the students learned what they were expected to do when contacting other health professionals. One participant said: “Calling the doctor, presenting a situation... It was unknown to me that I was expected to suggest what to do… I have been thinking about that after the simulation” (No. 4). Several communication tools – Identify, Situation, Background, Assessment and Recommendation (ISBAR) [20], as well as “closed loop” communication with repetition of the spoken message – were used during the scenarios. Using these communication tools in simulation prepared the students for clinical practice. One said: “I practiced closed loop during simulation. Then, you call the doctor for an ordination. I have brought the closed loop with me to the clinical practice” (No. 7).

Based on the simulation-based training, the participants also paid more attention to nurses’ personal responsibility and the responsibilities of the other staff involved in the situation. “Experiencing being a unit… and feeling what may happen to me if colleagues do not do their job. Then, I realized that I must be able to do my part of the job. That was very important” (No. 2).

The participants also noticed the importance of leadership and became more aware of the nurse’s role as a leader. One participant expressed: “After simulation, I have dared to be more of a leader… Feeling the responsibility of being the leading nurse in the scenario, delegating tasks to the other nurses” (No. 8).

The participants became more conscious of their role in relation to the patient’s relatives, and simulation made them more prepared to communicate with the relatives and meet their needs. As one participant said about the relatives, “It is important to include them because they know the patient best… They can have a lot of information that we do not know” (No. 7). Another participant said: “I really haven’t considered the relatives during my studies. There isn’t much focus on them, but during simulation we learned that we must take care of the relatives… Advise them if they want to leave or explain to them what is going on in the situation. It is really important” (No. 8).

## Discussion

The results showed that simulation-based training promoted self-confidence as well as improved clinical skills and judgement, and the participants discovered the importance of communication and team collaboration in a clinical context. That participants’ expressed increased self-confidence after performing simulation-based training in the clinical laboratory corresponds to the findings of Kimhi et al. [21], Cummings and Connelly [22] and Liaw et al. [23]. Smith and Roehrs [24] stated that when implementing simulation, the design elements of “objectives”, “support”, “problem solving” “guided reflection” and “fidelity” combine to contribute to increased self-confidence for nursing students and that “problem solving” contributes most of all to this increased self-confidence. Self-confidence is also dependent on the simulation facilitators’ ability to create an inclusive learning environment during the simulation sessions [15]. Self-confidence was described by the participants as very important to their learning outcomes. The facilitator’s abilities and professional knowledge were important, but equally important was their interest in and dedication to the students’ learning process [25]. Descriptions of a positive, encouraging attitude from the facilitator during the simulation-based training, especially during debriefing, corresponds in the interviews with students’ experiences of a positive change in attitude after the simulation-based training. The participants’ initial uncertainty and self-criticism were positively affected by peers acknowledging their performance and behaviour. They said that during simulation-based training, they were perhaps too focused on self-criticism and fear of making mistakes in front of their peers. The positive feedback from the facilitator and peers forced the participants to consider whether they were being fair to themselves.

Through the simulation-based training, the participants also became more aware of their own reactions to stress. The feedback they received from others gave them a better understanding of their own behaviour under pressure, and they also became more aware of their own bodily reactions. They brought this awareness with them into clinical practice.

Increased self-confidence, however, does not predict stronger clinical performance. According to Liaw et al. [23], it reveals the potential danger of over-confidence in one’s performance. This study revealed no examples of this, but we only included statements from the participants, not from the supervisors of the clinical practice. Supportive interviews with the supervisors could provide additional data on this issue.

The participants asserted that their examinations of clinical symptoms and vital signs in simulation-based training made them more assured in their decision-making in clinical situations. They felt more prepared for clinical placement having used decision-making tools, having learned to use technical and monitoring equipment, and having used ISBAR when performing communication procedures, e.g., calling the doctor for advice. These findings are similar to the themes raised by participants in a study by Buykx et al. [26], which found that assessment skills such as routine patient observations, emergency management skills, and personal attributes such as confidence and communication skills are important lessons.

The participants experienced many practical challenges in the high-fidelity scenarios. As a result of the simulation-based training, they discovered many important tasks and challenges not described in reading lists or in theoretical assignments. They experienced the value of simulation-based training in exposing unexpected aspects of clinical situations that were not described in their syllabus. One example was when they had to give a drug intravenously because the patient’s blood pressure was decreasing, and they discovered that the patient had no intravenous cannula (No. 6).

The importance of relevant scenarios was highlighted by many of the participants. This corresponds to the findings of Houghton et al. [27], who stated that authenticity in simulation-based training is crucial. The participants reported that the closer the scenarios were to reality, the easier it was for them to transfer their experiences from the simulation to their clinical placements. Ewertsson et al. [28] explained that in students’ experiences, there are often differences between the ways they have learned to perform practical skills in the clinical simulation laboratory and the ways they are supposed to perform them in clinical placements. These differences were perceived by the students as difficult and negative. However, Ewertsson et al. [28] suggested that these differences also can be seen as positive because they give the students further opportunities for reflection.

Performing as a team caused the participants to experience the necessity of communication, interaction and interdependence with each other to resolve different clinical situations appropriately. They became more aware of the importance of their personal knowledge and skills and of the importance of their leadership as nurses. The transfer of these experiences is also valuable. As Apker et al. [29], [p., 110] stated, “Nurses who identify the varying role expectations of team member constituencies and develop a repertoire of communicative strategies to manage those expectations may be better equipped to meet the multiple challenges presented in modern nursing roles”.

The participants also discovered how much they could benefit from communication and cooperation with the patient’s relatives. They described the relatives as a valuable source of information about the patient’s individual habits and desires. The nurses’ communication skills, as well as their ability to negotiate with the patient and include the patient and relatives in decision-making, were important aspects of nursing practice [30, 31]. In deteriorating patient situations, as in the two scenarios examined here, the participants expressed their uncertainty about how to meet the needs of the relatives: they were ambivalent about whether they should let them stay or ask them to leave the room. The participants’ detection of these issues resulted in engaged reflections during the debriefing sessions. The participants reported that these issues were barely referred to in their courses and that they transferred their experiences from the simulation-based training to clinical practice, paying more attention to the patients’ relatives than they had in earlier clinical practice.

The participants’ experienced from simulation-based training remained as enduring and conscious learning outcomes throughout their completion of clinical practice. Weeks after the simulation training, the participants still clearly remembered much of their personal and professional learning from the simulation-based training. The participants’ dialogues in the focus groups were rich and reflexive about their experiences in the simulation-based training and how they had transferred these experiences to their clinical placements. O’Donnell et al. [32] stated that further research should measure the retention of learning, learning decay and the transferability of knowledge and skills. This study does not measure these elements; however, our findings indicate that simulation-based training promotes enduring learning in various areas.

Some participants, however, argued that placing the simulation training first in the semester or late in the semester after exams was unfavourable. The organization and implementation of the simulation program during the semester was important for the level of the students’ learning outcomes and ability to transfer their learning to clinical placements. Participants who played an active role in the scenarios had better learning outcomes than did those participants who were only observers. Ewertsson et al. [28] confirmed this, noting that students’ learning is enhanced when they are actively involved in gaining knowledge through experience with problem solving and decision-making.

One could make the criticism that the facilitators did not implement the simulation scenarios in a similar manner. To increase the students’ learning outcomes, it is necessary to perform the scenarios more than once [22, 28]. Repeating the scenarios may demand more resources, but it might be possible to organize the simulation sessions better to achieve more effective simulation-based training.

The combination of more high-fidelity simulations with conventional clinical experiences is a more effective educational strategy according to Curl et al. [33]. The results of this study enhance our understanding of simulation-based training for nursing students, showing this training to be a multimodal source that helps students transfer their learning outcomes to clinical practice. To enhance the transference of the learning outcomes from simulation to clinical practice, close collaboration between higher education institutions and clinical placements is recommended [27].

### Methodological considerations

The 32 participants represented a distribution of demographic characteristics quite similar to the distribution of nursing students in the bachelor program at the University. Both active performers and observers from the simulation sessions were represented in the focus group interviews. For known reasons, there are more citations from some focus groups than others. In the interviews completed in May 2016, the students’ experiences were mostly related to the lack of information about the simulation program. In later interviews, the information was improved, and the participants reported more experiences from the simulation training and the transfer to clinical placements. This experience emphasises the importance of the organization and implementation of the simulation.

## Conclusions

The use of simulation-based training has increased and become an integrated part of nursing education. This study revealed students’ transfer of their learning from simulation-based training to clinical practice; they increased their self-confidence, skills and clinical judgement, as well as their understanding of the importance of communication and team cooperation. Experiences from simulation-based training remain as enduring and conscious learning for the students through their completion of clinical practice. The organization of the program and its implementation in the curriculum are crucial for both learning outcomes and the extent of the transfer of learning to clinical practice.

### Recommendations

Simulation-based training integrated into the bachelor program in nursing is a recommended preparation for clinical practice. Future studies should focus on observations of the nursing students’ clinical performance and on evaluations from the supervisors of nursing students who have performed simulation-based training before clinical practice to understand more about the transfer of learning from simulation-based training to clinical practice. Research on collaborative experiences between higher education institutions and health services can also contribute to deepening the understanding of the role of simulation-based training in nursing education.

## Data Availability

The datasets collected in this study are available from the corresponding author on reasonable request.

## References

[1] Rothgeb MK (2008). Creating a nursing simulation laboratory: a literature review. J Nurs Educ.

[2] Hegland PA, Aarlie H, Stromme H, Jamtvedt G (2017). Simulation-based training for nurses: systematic review and meta-analysis. Nurse Educ Today.

[3] Harder BN (2010). Use of simulation in teaching and learning in health sciences: a systematic review. J Nurs Educ.

[4] Yuan HB, Williams BA, Fang JB, Ye QH (2012). A systematic review of selected evidence on improving knowledge and skills through high-fidelity simulation. Nurse Educ Today.

[5] Howard VM, Englert N, Kameg K, Perozzi K (2011). Integration of simulation across the undergraduate curriculum: student and faculty perspectives. Clin Simul Nursing.

[6] Shin S, Park JH, Kim JH (2015). Effectiveness of patient simulation in nursing education: meta-analysis. Nurse Educ Today.

[7] Sundler AJ, Pettersson A, Berglund M (2015). Undergraduate nursing students' experiences when examining nursing skills in clinical simulation laboratories with high-fidelity patient simulators: a phenomenological research study. Nurse Educ Today.

[8] Cooper A (2015). High-fidelity simulation for neonatal nursing education: an integrative review of the literature. Neonatal Netw.

[9] Cant RP, Cooper SJ (2010). Simulation-based learning in nurse education: systematic review. J Adv Nurs.

[10] Houghton CE, Casey D, Shaw D, Murphy K (2012). Staff and students' perceptions and experiences of teaching and assessment in clinical skills laboratories: interview findings from a multiple case study. Nurse Educ Today.

[11] Houghton CE, Casey D, Shaw D, Murphy K (2013). Students' experiences of implementing clinical skills in the real world of practice. J Clin Nurs.

[12] Cates LA, Wilson D (2011). Acquisition and maintenance of competencies through simulation for neonatal nurse practitioners: beyond the basics. Adv Neonatal Care.

[13] Chamberlain J (2017). The impact of simulation prebriefing on perceptions of overall effectiveness, learning, and self-confidence in nursing students. Nurs Educ Perspect.

[14] Kirkman T, Hall C, Winston R, Pierce V (2018). Strategies for implementing a multiple patient simulation scenario. Nurse Educ Today.

[15] Nash R, Harvey T (2017). Student nurse perceptions regarding learning transfer following high-fidelity simulation. Clin Simul Nursing..

[16] Kitzinger J (1995). Qualitative research. Introducing focus groups. BMJ.

[17] Krueger RA (2014). Focus groups: a practical guide for applied research.

[18] Gibbs G (1988). Learning by doing. A guide to teaching and learning methods.

[19] Braun V, Clarke V (2006). Using thematic analysis in psychology. Qual Res Psychol.

[20] The Government of South Australia. ISBAR - Identify, situation, background, assessment and recommendation. 2018. https://www.google.com/search?q=isbar+communication+tool&ie=utf-8&oe=utf-8&client=firefox-b. Accessed 5 Feb 2018.

[21] Kimhi E, Reishtein JL, Cohen M, Friger M, Hurvitz N, Avraham R (2016). Impact of simulation and clinical experience on self-efficacy in nursing students: intervention study. Nurse Educ.

[22] Cummings CL, Connelly LK (2016). Can nursing students' confidence levels increase with repeated simulation activities?. Nurse Educ Today.

[23] Liaw SY, Scherpbier A, Rethans JJ, Klainin-Yobas P (2012). Assessment for simulation learning outcomes: a comparison of knowledge and self-reported confidence with observed clinical performance. Nurse Educ Today.

[24] Smith SJ, Roehrs CJ (2009). High-fidelity simulation: factors correlated with nursing student satisfaction and self-confidence. Nurs Educ Perspect.

[25] Tosterud R, Hall-Lord ML, Petzäll K, Hedelin B (2014). Debriefing in simulation conducted in small and large groups - nursing students’ experiences. J Nursing Education Pract.

[26] Buykx P, Kinsman L, Cooper S, McConnell-Henry T, Cant R, Endacott R, Scholes J (2011). FIRST2ACT: educating nurses to identify patient deterioration - a theory-based model for best practice simulation education. Nurse Educ Today.

[27] Houghton CE, Casey D, Shaw D, Murphy K (2013). Educational issues in nursing practice. J Clin Nurs.

[28] Ewertsson M, Allvin R, Holmstrom IK, Blomberg K (2015). Walking the bridge: nursing students' learning in clinical skill laboratories. Nurse Educ Pract.

[29] Apker J, Propp KM, Zabava Ford WS (2005). Negotiating status and identity tensions in healthcare team interactions: an exploration of nurse role dialectics. J Appl Commun Res.

[30] Baillie L (2016). Working in partnership with patients and carers. Nurs Stand.

[31] Campinha-Bacote J (2011). Delivering patient-centered care in the midst of a cultural conflict: the role of cultural competence. Online J Issues Nurs.

[32] O'Donnell JM, Decker S, Howard V, Levett-Jones T, Miller CW (2014). NLN/Jeffries simulation framework state of the science project: simulation learning outcomes. Clinical Simulation in Nursing..

[33] Curl ED, Smith S, Ann Chisholm L, McGee LA, Das K (2016). Effectiveness of integrated simulation and clinical experiences compared to traditional clinical experiences for nursing students. Nurs Educ Perspect.

